# Supplementary material for: Treatment with Angiotensin-(1-7) Prevents Development of Oral Papilloma Induced in K-ras Transgenic Mice
Source: Int J Mol Sci. 2022 Mar 26;23(7):3642. doi: 10.3390/ijms23073642 (PMC8998511; doi:10.3390/ijms23073642)
Supplement: Supplementary file 1 [file ijms-23-03642-s001.zip › ijms-1635133-supplementary.pdf]

Supplementary Figure S1

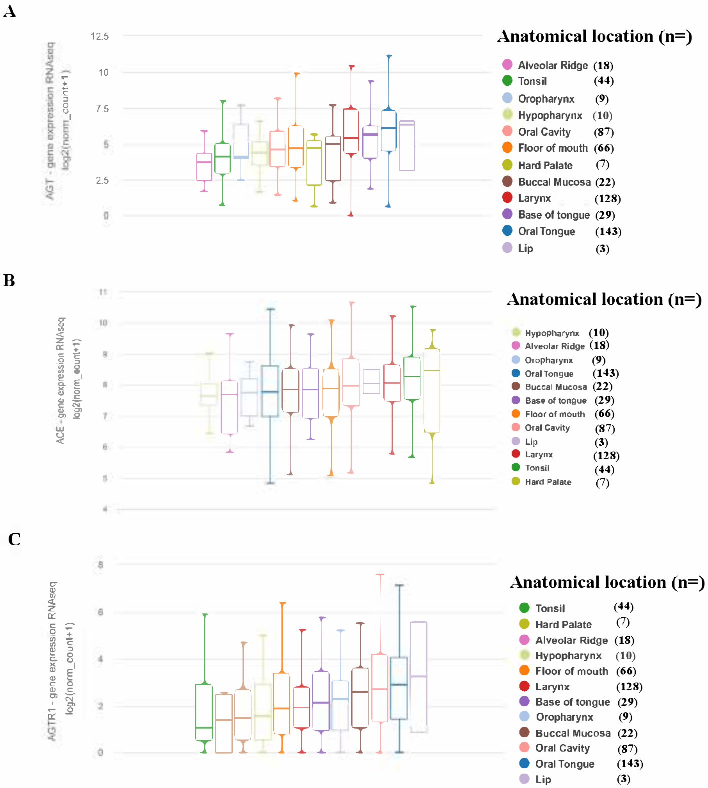

Supplementary Figure S1. Levels of gene expression of members of the classical components of the renin-angiotensin system in head and neck squamous cell carcinoma (HNSCC). Data from the TCGA HNSCC study (21 datasets, n=604 samples, 566 RNA sequenced) was used to investigate the expression of angiotensinogen (AGT) (A), ACE (B) and angiotensin receptor type 1 AT1 (AGTR1) (C) along distinct anatomic locations of HNSCC. Analyses were performed with the UCSC Xena browser.
